# Smoke Rings: Towards a Comprehensive Tobacco Free Policy for the Olympic Games

**DOI:** 10.1371/journal.pone.0130091

**Published:** 2015-08-07

**Authors:** Kelley Lee, Gary Fooks, Nathaniel Wander, Jennifer Fang

**Affiliations:** 1 Faculty of Health Sciences, Simon Fraser University, Burnaby, BC, Canada; 2 School of Languages and Social Sciences, Aston University, Birmingham, United Kingdom; 3 Cayo Pájaro Chico, Ranchito, Corozal, Belize; The National Institute for Health Innovation, NEW ZEALAND

## Abstract

**Background:**

The tobacco industry has long sought affiliation with major sporting events, including the Olympic Games, for marketing, advertising and promotion purposes. Since 1988, each Olympic Games has adopted a tobacco-free policy. Limited study of the effectiveness of the smoke-free policy has been undertaken to date, with none examining the tobacco industry’s involvement with the Olympics or use of the Olympic brand.

**Methods and Findings:**

A comparison of the contents of Olympic tobacco-free policies from 1988 to 2014 was carried out by searching the websites of the IOC and host NOCs. The specific tobacco control measures adopted for each Games were compiled and compared with measures recommended by the WHO Tobacco Free Sports Initiative and Article 13 of the Framework Convention on Tobacco Control (FCTC). This was supported by semi-structured interviews of key informants involved with the adoption of tobacco-free policies for selected games. To understand the industry’s interests in the Olympics, the Legacy Tobacco Documents Library (http://legacy.library.ucsf.edu) was systematically searched between June 2013 and August 2014. Company websites, secondary sources and media reports were also searched to triangulate the above data sources.

This paper finds that, while most direct associations between tobacco and the Olympics have been prohibited since 1988, a variety of indirect associations undermine the Olympic tobacco-free policy. This is due to variation in the scope of tobacco-free policies, limited jurisdiction and continued efforts by the industry to be associated with Olympic ideals.

**Conclusions:**

The paper concludes that, compatible with the IOC’s commitment to promoting healthy lifestyles, a comprehensive tobacco-free policy with standardized and binding measures should be adopted by the International Olympic Committee and all national Olympic committees.

## Introduction

The Olympic Games are one of the world’s premier sporting events. As well as celebrating sporting excellence, the Games aim to uphold “a philosophy of life, exalting and combining in a balanced whole the qualities of body, will and mind” [1]. Based on a belief that amateurism is central to these ideals, the International Olympic Committee (IOC) long resisted direct involvement with commercial interests. Financing problems from the mid 1970s, however, led to a major expansion of corporate sponsorship and commercialisation under President Juan Antonio Samaranch [2,3,4]. For private companies, the breadth of sporting events, number of participating countries and worldwide audience were powerful incentives to be associated with the Olympics. By 2013 the Olympics were ranked the world’s second most valuable sporting event brand after the National Football League’s Super Bowl [5].

The tobacco industry’s commercial links with the Olympics date from at least the 1930s but, in the 1980s, this association began to be questioned. In 1988, the Canadian National Olympic Committee (NOC) designated the Calgary Winter Games as the first “Smoke-Free” Olympics, banning tobacco sponsorship and introducing non-smoking areas. The IOC and host NOCs have adopted a tobacco-free policy for every summer and winter Games since held. In July 2010, promotion of the Olympics as “tobacco free” was emphasised in an agreement between the World Health Organization (WHO) and IOC [6].

Analyses of the Olympic Games and public health to date have focused on health planning for mega-events including medical services [7], disease risks [8], accidents and injuries, emergency response and security [9,10,11,12]. The potential for the Olympics to create a health legacy, from increased physical activity [13,14,15] or improved environment [16] and public health infrastructure [17], has also been examined. Limited study of the Olympic tobacco free policy has been undertaken to date, with one study of the Barcelona Summer Games [18], rather than over time. None examine the tobacco industry’s involvement with the Olympics.

This paper reviews the tobacco-free policies adopted by each Olympic Games since 1988. It is argued that, while most forms of direct association with tobacco have now been effectively prohibited, a variety of indirect associations continue to undermine the tobacco-free policy. It is argued that this is due to variation, across different Games and national Olympic committees, in the scope of the policies adopted, and continued efforts by the tobacco industry to associate with Olympic ideals. The findings suggest the need for comprehensive restrictions, as prescribed under the WHO Framework Convention on Tobacco Control (FCTC), to be adopted by the IOC and all national Olympic committees. This paper puts forth a proposed set of principles, compatible with the IOC’s commitment to promoting healthy lifestyles, to make the Olympic Games truly tobacco free.

## Methods

A comparison of the contents of Olympic tobacco-free policies from 1988 to 2014 was carried out by searching the websites of the IOC and host NOCs. The specific tobacco control measures adopted for each games were compiled and compared with measures recommended by the WHO Tobacco Free Sports Initiative and Article 13 of the Framework Convention on Tobacco Control (FCTC) [19]. A small number of semi-structured interviews were carried out with key informants involved with the adoption of tobacco-free policies for Seoul, Vancouver and London, and WHO officials in relation to Beijing and Sochi. Subjects were recruited through professional networks of the authors who, while not a representative sample of all the games analyzed, provided supplemental insights into challenges faced during the adoption of tobacco free policies for selected games. To understand industry’s interests in the Olympics, the Legacy Tobacco Documents Library (http://legacy.library.ucsf.edu) was systematically searched between June 2013 and July 2014, combining the keyword “Olympic*” using Boolean terms with host cities, athlete names and sporting events. This initial search of the collection, comprising documents dating from the early twentieth century to 2000s, yielded 4,160 documents of which 328 were deemed most relevant to activities since 1988. Company websites, secondary sources and media reports were searched using the same keywords to identify interests with the Olympics during any time which helped to triangulate the above data sources.

This research received ethics approval from the Office of Research Ethics (ORE), Simon Fraser University (File number 2012s0556). All key informants provided written consent to participate in this study using consent forms approved by the ORE. The authors have completed the NIH Office of Extramural Services web-based training course “Protecting Human Research Participants.”

## Results

### Olympics as global tobacco “marketing platform” [20]

While sports sponsorship and tobacco date from 1875 [21], specific association with the Olympics began around the 1930s with the endorsement of cigarette brands by medal athletes such as swimmer Art Lindegren [22], “Speed Queen of the Olympics” skater Kit Klein [23], and sprinter Jesse Owen [24,25]. This commonplace practice remained the primary focus until the 1960s when tobacco companies began to support events deemed to have desirable qualities such as risk, glamour and excitement [26] such as downhill skiing [27], ice dancing [28] and windsurfing [29]. From the 1970s, under the “intensifying commercialization and commodification of the Olympic product” [30], companies were invited to become official sponsors of the Games as a whole. The IOC offered the Olympics as “one of the most effective international marketing platforms in the world”[31]. Amid growing restrictions on other forms of tobacco marketing [32], tobacco companies saw sponsorship as highly attractive given its “universal appeal” [33]. By the 1980s, British American Tobacco (BAT) deemed the Olympics second only to Formula One motor racing as an effective sports-based “marketing platform” [20].

Public health advocates and athletes began to speak out concertedly against tobacco sponsorship of the Olympics in the 1980s [34]. In 1984, two Canadian skiers turned down prizes sponsored by a tobacco company [35]. Soon after, members of the Canadian Ski Association, supported by the Canadian Non-Smokers Rights Association [36] and Canadian Cancer Society, threatened to boycott the season “if RJR-Macdonald Tobacco Company is not replaced as a sponsor”, although the association voted against finding a new sponsor [37]. US diver Greg Louganis testified in 1988 to the US Congress that his dependence on a training facility funded by Philip Morris (PM), to prepare for the 1984 Moscow Summer Games, was the key factor in declining to chair the American Cancer Society’s annual campaign challenging smokers “to stop using tobacco” [38]:

I had become a slave to a tobacco company…. Philip Morris representatives made it very clear that if I continued to speak out nationally [about tobacco and health], my career at, and association, with Mission Viejo [Realty Group, a PM subsidiary] would be over [39].

It was in this context that the organisers of the 1988 Calgary Winter Games announced the first “smoke-free” Olympics. The IOC adopted its own tobacco-free policy shortly afterwards:

Sports is about health. We firmly believe that the Olympics should not be associated with unhealthy behaviours, that’s why we work so hard to promote policies such as the tobacco-free Olympics. We can promote many such healthy lifestyles and are actively working with WHO in drafting similar policies [40].

### Comparative analysis of Olympic tobacco free policies, 1988–2014

Since 1988, all 15 summer and winter Olympics have adopted a smoke or tobacco-free policy which collectively span 11 specific measures (Table 1). A comparative analysis of the measures adopted shows that two—prohibitions on tobacco advertising in Olympic venues and designated smoking areas—have been adopted by all 15 games. The prohibited acceptance of tobacco companies as official sponsors of the games has been adopted by 14 events. The adoption of smoke-free indoor and outdoor venues has been adopted by 13 and 12 events respectively. The least frequent measures, adopted by five games, have been smoke-free restaurants and bars, smoke-free Olympic transport and anti-smoking messages or displays.

**Table 1:** Measures contained in tobacco-free policies adopted by Summer and Winter Olympics since 1988.

Olympic Games and year	Prohibit acceptance of tobacco sponsorship	Prohibit tobacco advertising	Prohibit distribution and sale of tobacco products	Smoke-free indoor venues	Smoke-free outdoor venues	Smoke-free Olympic Village	Smoke-free restaurants and/or bars	Smoke-free Olympic transport	Designated smoking areas	Anti-smoking messages or displays	Public health campaigns
**Calgary Winter, 1988** ^**41**^	✓	✓	✓	✓	✓			✓	✓		
**Seoul Summer, 1988**	✓	✓	✓						✓		
**Albertville Winter, 1992 [42,43]**	✓	✓		✓	✓	✓			✓		
**Barcelona Summer, 1992 [44]**	✓	✓	✓	✓	✓	✓		✓	✓	✓	✓
**Lillehammer Winter, 1994 [45]**		✓	✓	✓		✓	✓	✓	✓	✓	
**Atlanta Summer, 1996**	✓	✓	✓	✓	✓			✓	✓		✓
**Nagano Winter, 1998 [46]**	✓	✓		✓	✓	✓			✓		
**Sydney Summer, 2000 [47]**	✓	✓	✓	✓	✓				✓	✓	✓
**Salt Lake City Winter, 2002 [48]**	✓	✓	✓	✓	✓	✓	✓		✓		✓
**Athens Summer, 2004 [49]**	✓	✓				✓	✓	✓	✓		✓
**Turin Winter, 2006 [50]**	✓	✓		✓	✓	✓			✓		
**Beijing Summer, 2008 [51]**	✓	✓	✓	✓	✓				✓	✓	✓
**Vancouver Winter, 2010 [52]**	✓	✓	✓	✓	✓	✓	✓		✓		
**London Summer, 2012 [53,54]**	✓	✓	✓	✓	✓	✓			✓		
**Sochi Winter, 2014 [55]**	✓	✓	✓	✓	✓	✓	✓		✓	✓	

A comparative analysis of games shows that none have adopted all 11 measures. The 1992 Barcelona Summer Games adopted the highest number (10), followed by 2002 Salt Lake City (9) and 2014 Sochi (9) Winter Games, while 1988 Seoul Summer Games (4) adopted the lowest number. The latter focused on designating all inside Olympic venues and meetings as smoke free and was considered at the time, given little precedence, an important achievement [56]. There was no clear pattern over time regarding games and specific measures adopted. The average number of measures adopted 7.4 measures.

This analysis shows that, while Olympic Games have been designated as smoke or tobacco-free since 1988, the interpretation of what this means has varied significantly. A search of the IOC website and materials finds that, beyond a statement in *The Olympic Marketing Fact File 2014*, that the IOC “does not accept commercial associations with tobacco products,” there is no official tobacco-free policy which sets out the specific measures to be adopted. Instead, it is left to each national Olympic organizing committee to adopt and specify its own policy. It is this delegation of authority which explains the variation in the scope of measures adopted as shown in Table 1.

Semi-structured interviews conducted with public health individuals involved in the drafting of tobacco free policies, related to selected games for which key informants could be identified, suggest resistance by different interests to strong and comprehensive measures. For the 2010 Vancouver Winter Games, for example, the vision for a “completely tobacco free” games set out by Vancouver Coastal Health Authority was initially supported by VANOC. However, a few months before the start of the games, pressure by the IOC and other national Olympic committees, that strict measures would “unrealistic and unreasonable” for foreign visitors unused to stringent North American public smoking bans, led VANOC to temper the policy. Smoking areas were subsequently permitted on all Olympic sites including the athletes village and sporting venues [57]. Similarly, as part of initial plans to create a health legacy from the 2012 London Summer Games, regional officials of the National Health Authority proposed a more comprehensive tobacco-free rather than smoke-free policy to LOCOG. As well as being unaware of the IOC policy, officials found LOCOG staff believed tobacco use was a “personal choice” and thus unsupportive of strong tobacco control. The election of a coalition government in the UK in 2010, and disbanding of the London Regional Tobacco Control Team amid public sector cuts, further weakened commitment. The London games were eventually designated as smoke-free with “discrete smoking areas” [58]. This paper argues that, as well as a lack of clarity and consistency in what the Olympic tobacco free policy means, and how it has been adopted for specific games, internal documents suggest that since 1988, this variation has allowed tobacco companies to use “indirect ways to tie into Olympic excitement” [59].

### Sponsorship of Olympic broadcast coverage

Documents suggest tobacco companies sought to sponsor Olympic broadcast coverage where no restrictions have been adopted nationally. In Malaysia, BAT brand Peter Stuyvesant sponsored broadcasts of the 1996 Atlanta Summer Games. While the IOC protested to Radio Television Malaysia, that no tobacco trademark should be associated with the Olympics, and that BAT’s advertising campaign falsely implied the brand was an official sponsor, it stopped short of withdrawing broadcasting rights [60,61]. In Myanmar, where the “anti-tobacco lobby…is very limited”, during the 1990s BAT worked through a public relations company to supply “high quality television programming…such as the SEA [Southeast Asian] Games and Olympics” [62]. With many countries adopting restrictions on tobacco sponsored broadcasts, to adhere to the FCTC, the potential for crossborder communications to circumvent national restrictions attracted industry interest. Crossborder broadcasts have been an important industry strategy for Formula One racing [63]. For the Olympics, the popularity of internet-based “push technology” led BAT to explore sponsorship of a website similar to “PointCast’s Summer Games channel” [64]. These plans were later abandoned as interest in push technology waned [65]. Recognising the problem of crossborder broadcasts, the European Union adopted the *Tobacco Advertising Directive* (2003/33/EC) which enacted an EU-wide ban on crossborder tobacco advertising and sponsorship using print media, radio and the internet, and covers events involving multiple member states such as the Olympics [66]. Where national and regional restrictions do not exist, tobacco sponsorship of Olympic broadcasts remains possible.

### Advertising in Olympic- themed print media

While all games since 1988 have banned all forms of tobacco advertising in Olympic venues, the current Olympic tobacco-free policy does not extend to Olympic-themed publications, not even by official sponsors. Tobacco companies have thus created associations with the Olympics through themed print media. This has been most evident in special issues, commemorative editions and guides to the Olympics of popular magazines published in the US where a ban on tobacco advertising was ruled unconstitutional under the First Amendment in 2001 [67]. For example, RJR Nabisco purchased daily space in the sports section of *USA Today* for the whole of 1988 to “showcase several RJ Reynolds Tobacco USA brands, particularly those with sports marketing programs” [50]. During both summer and winter games, the ads featured “products that are official sponsors of the U.S. Olympic Team” [68]. During the 1996 Atlanta Summer Games, *Southern Living* offered RJR one page in the “Commemorative Guide to the Olympic South” [69]. For the same games, PM contracted to place a Benson & Hedges ad on the back cover of a souvenir program guide to Olympic basketball games [70]. AOL Time Warner (an Official Worldwide Sponsor since 1982), has long solicited tobacco advertising [71]. During Olympic years, *Sports Illustrated* carried full-page tobacco advertising for Olympic preview issues, guides, commemorative editions until 2008 [72,73,74,75,76,77]. Olympic editions of *Newsweek*, published by Washington Post Incorporated, similarly carried full-page advertisements by Japan Tobacco International (JTI) and BAT [78]. Strongly criticised by public health advocates [79], Time Warner and the Washington Post adopted “selective binding” in 2005 to remove tobacco advertising in magazines delivered to American schools [80]. While the magazine ad business is reportedly in decline, it is reported that the number of tobacco ads in US magazines rose by 11% in 2012 due to increased restrictions on other forms of advertising (e.g. broadcast media) [81].

### Advertising to Olympic travellers

Documents suggest tobacco companies looked for “new communication techniques” to advertise to the “large increases in passengers of the key nationalities” travelling to games [82]. Most international airports have remained exempt from tobacco advertising bans, to facilitate duty free sales, and are thus considered “one of the last remaining havens for the tobacco industry” [83]. Visitors travelling to the Olympics have remained exposed to targeted messaging in the form of billboards, industry-sponsored smoking rooms, and point-of-sale advertising since 1988. For example, PM exclusively sponsored the installation of eight glass-enclosed smoking lounges at Hartsfield International Airport in Atlanta in 1995, complete with “state-of-the-art ventilation systems”[84]. As a part of an “Accommodation Program” (see below), the lounges were “recognizable primarily by Marlboro signage, and secondarily through signage from The Accommodation Program, a distinctive circular yin-yang symbol which tell customers that both non-smokers and smokers are welcome” [65]. The lounges were described as providing “a smoke-free public environment for our non-smoking flyers while offering a comfortable lounge setting for our smoking flyers without forcing them out the door” [65]. Documents describe sales increasing five-fold, to one hundred packs a day from the previous twenty [85]. After the Olympics, the advertising was removed [86].

### ‘Accommodation’ of Olympic visitors in host cities

As described elsewhere, since the 1990s the industry-led Accommodation Program has been deployed worldwide to weaken public smoking restrictions [87]. Instead of banning smokers, the industry has argued for the creation of smoking areas in hotels, restaurants and other venues. It is argued here that, with the adoption of smoke-free indoor and outdoor Olympic venues, PM used the rationale of “accommodating” Olympic visitors to market and promote smoking. In 1995 PM promoted the accommodation of smokers and non-smokers in hotels, restaurants and other public places, where Olympic visitors congregate, as a gesture of hospitality. Internally, however, PM described the program as offering “new advertising opportunities” [88]. Gaining endorsement from the Atlanta Convention and Visitors Bureau [89], the city’s hospitality industry was convinced to roll “out the red carpet for the millions of smoking and non-smoking guests attending the summer Olympics”, by successfully encouraging 25% of restaurants to increase the size of their smoking areas [90]. Designating Atlanta a “Model City of Accommodation” [91], the scheme was heavily promoted with signage in almost 1000 businesses to “heighten awareness of the Philip Morris Accommodation program” among visitors [92]. Visibility was further enhanced by “Welcome Stations and Information Booths” located at key public transport points, offering visitors guides to local amenities with smoking areas [93]. In this way, PM sought “to leverage the Olympics as a visible platform of the Program’s ability to help operators accommodate international visitors” [72], targeting “visitors to the Atlanta 1996 Summer Olympics, particularly international visitors who smoke” [94]. All visitors, smoking and non-smoking, were greeted with tobacco advertising in restaurants, bars, hotels, taxis, public transport, bus shelters, convenience stores and “temporary kiosks” near to the Olympic Ring [41,95]. Outside of Olympic venues, where sales of tobacco products remain permitted, special displays were set up using branded banners, neon signs and promotional materials such as T-shirts [96].

The accommodation of smokers proved to be an argument to weaken public smoking bans at other games. For example, French organisers distributed ashtrays at the 1992 Albertville Winter Games despite the policy that “all Olympic venues will be no smoking areas–with the exception of a small number of smoking areas” [97]. Organisers of the 1992 Barcelona Summer Games distributed disposable lighters to journalists in information kits [98], and it was later observed that the ban on tobacco advertising and smoking at the Barcelona games was “largely ignored” [99]. At the 2004 Athens Summer Games, the UN Environment Programme and Athens Organising Committee distributed 40 000 portable paper ashtrays to the 10 000 journalists attending the games: “We certainly don’t want to be seen as promoting smoking, but since we know it is not possible to stop all journalists from lighting up” [100]. At the 2010 Vancouver Winter Games it was initially announced that Vancouver would be completely smoke-free, with no designated smoking areas, a policy strongly supported by the Vancouver Organizing Committee (VANOC). The IOC and other NOCs, however, argued that visitors, coaches, officials and journalists from countries with less stringent smoking restrictions needed accommodation [57]. Under pressure from the IOC, VANOC changed the policy in late 2009, from a complete ban to the installation of 25 designated smoking areas outside of indoor venues [101]. The change drew local criticism given the adoption of the Tobacco-Free Sports Initiative by the BC Ministry of Health in 2003 with the goal of increasing the number of sporting organizations and facilities with Tobacco-Free Sport leading up to the 2010 Olympics [102]. In London, a draft tobacco-free policy by public health officials called on the London Organising Committee of the Olympic and Paralympic Games (LOCOG) to adopt a complete smoking ban. However, LOCOG viewed a ban as an unreasonable infringement on personal freedom of visitors, particularly smokers [58]. Because few internal documents date from 2003 onwards, it is not possible to determine whether the industry was involved in these policy decisions. These findings suggest that the lack of clear agreement, on the definition of a smoke-free public place under the Olympic tobacco-free policy, leaves the policy open to industry-supporting accommodation arguments.

### Thematic advertising and implied allusion

Ambush marketing occurs when “a company that has no formal rights as an official sponsor, associates its own brand with a sport event with the intent of communicating the false impression that it is a sponsor” [103]. The practice has become prevalent during Olympic Games, and organisers have sought legislative remedies focused on protecting financial interests [104,105,106]. Thematic advertising is a form of ambush marketing by which official symbols are not used directly, but in such a way as “to give the impression that [the tobacco company] is officially related to the event” [87]. This was common before 1988 through the use of special promotions and giveaways of Olympic-related goods such as guidebooks, replica medals, posters and score sheets. The Rothman’s “Help Win Medals for Britain” campaign of 1972, for example, sought to foster a “feeling of patriotic well-being from contributing by proxy to the British Olympic Appeal Fund”. In 1984, RJR branded and inserted Olympic scoresheets in the *Los Angeles Times* and at US retail outlets [107].

Documents suggest these practices continued after 1988. For the 1988 Seoul Summer games, KT&G (then called Korean Tobacco & Ginseng) introduced the “Eighty Eight” cigarette brand, which continues to be sold today, to celebrate “the national pride of Seoul’s hosting of the 1988 Olympics” [108]. After considering the launch of an “Official Cigarette of the ‘96 Olympics”, with names such as Torch, Flame, Gold and Gold Medal [109], RJR instead held a 2-pack lighter promotion of Salem [110] and a Camel t-shirt promotion [111]. In 2000 BAT used the slogan “Go for Gold” to link the gold packaging of Benson & Hedges to the Sydney Summer Games [112]. During the 2008 Beijing Summer Games, numerous special edition cigarette brands were launched by Chinese tobacco companies using Olympic symbols (For a list of Chinese special edition Olympic cigarettes see http://www.zigsam.at/C_ChinaSE.htm). The torch relay also prominently featured workers and executives of tobacco companies claiming affinity to Olympic ideals [113,114].

The organising of branded attractions near Olympic venues has also been used as a means of thematic advertising. Prior to the 1998 Nagano Winter Games, RJR organized themed bar nights at local venues which exclusively sold Vantage cigarettes, and featured “teams” competing for “Gold”, “Silver”, and “Bronze” medals “in keeping with the Olympic spirit” [115]. RJR promoted *Camel* cigarettes during the 2002 Salt Lake City Winter Games by paying bar owners US$3000 each to hold “Bar Nights”. The company supplied branded neon signs, matches, ashtrays, napkins and coasters, and paid for advertising in local newspapers. In return, bars sold Camel brands exclusively and displayed branded items prominently. Fearing that RJR was “proactively securing positioning for control of Bar Nights at every popular night spot in the Salt Lake/Park City area during the 2002 Olympics” [116], PM extended its “Marlboro Bar Nights” program [117] as “a special initiative”, including the distribution of branded CDs and other merchandise [118,119].

### Use of the Olympics to promote industry-sponsored youth anti-smoking campaigns

The disingenuous nature of industry-sponsored youth smoking prevention campaigns has been discussed elsewhere [120]. It is argued here that, from the late 1990s, tobacco companies used concerns about youth smoking to create indirect associations with the Olympics. The criticism of Chinese Olympic gold medallist Liu Xiang, as an “inappropriate” corporate ambassador for the Baisha Tobacco Group [121,122] reflects the shift in public opinion against tobacco product endorsement by Olympic athletes. While vowing to no longer use Olympians to promote tobacco use [123,124] instead companies recruited medalists for youth anti-smoking campaigns. PM, RJR, Rothmans and Reemstma were listed on sixty billboards in Moscow promoting the ““Smoking? No time for it!” billboard campaign during the 1998 World Youth Games [125] (succeeded by the Youth Olympic Games) for athletes 17 years and under. In 2001, with the Sporting Goods Manufacturers Association (SGMA), PM developed a Sports Development Program to Prevent Youth Smoking in 2001, with the goal to “provide additional and/or upgraded sports and physical fitness opportunities to youth” through partnering with various youth sports organizations including the National Council of Youth Sports and the US Olympic Committee [126]. In 2003 RJR produced a video, featuring US gold medalist Marion Jones, for distribution to “educators to use with students to…help them resist peer pressure to smoke” [127]. The same year Lorillard appointed US Olympians Mary Lou Retton (gymnastics) [128] and Kelly Clark (snowboarding) as spokespersons for its Youth Smoking Prevention Program. Clark promoted the “Wipe Out Teen Smoking” contest which rewarded contestants “who take a nonsmoking pledge at our website with decals and a chance to win one of 10 autographed snowboards” and a personal snowboarding lesson [129,130]. Advertisements for the contest appeared on youth-oriented programming such as "Buffy the Vampire Slayer" and "ESPN Action Sports and Music Awards," and magazines like *Sports Illustrated for Kids* [131].

### Sponsorship of Olympic athletes and teams

While the tobacco-free Olympic policy prohibits official sponsorship of games by tobacco companies, the ban does not extend beyond the events themselves. Indeed, recognising the high cost of developing competitive athletes and teams, the IOC removed the word “amateur” from the Olympic Charter in 1974. While the Charter governs what athletes may wear on their uniforms during the Olympic Games, it is silent on athlete sponsorship, leaving the issue of tobacco sponsorship to NOCs and sports governing bodies [132].

This paper finds many athletes, NOCs and sports federations, especially in the developing world [133], continued to accept tobacco sponsorship and other forms of funding after 1988. In the US, the Olympic Team received funds in 1990 through RJR’s Olympic Bingo, a promotional event distributing point of sale game cards and winnings [134]. The US Olympic Dinner, an annual fundraising event, was supported by the tobacco industry, with PM becoming involved at its initiation in 1991 [135], and at least two further occasions in 1994 [136] and 1996 [137]. In 1992, upon PM’s request, the Tobacco Institute sponsored a $10,000 table to the event [138]. In 1996, RJR hosted the entire Italian Olympic Team to train in Winston-Salem prior to the Atlanta Games, “light[ing] the top of the Reynolds Building in red, green and white” as a sign of welcome [139].

In Europe, then BAT Chairman Patrick Sheehy described BAT donating £46 000 from 1983 to 1995 because remarkably “the BOC [British Olympic Committee] probably managed not to realise that B.A.T. Industries is a tobacco company” [140]. Through its subsidiaries, BAT also sponsored athletes further afield. In 1992 BAT Kenya initiated “A Shilling for a Tree Olympics Campaign” launched by gold medallist Kip Keino (Vice President of the Kenya Olympic Committee), as “the BAT way of helping to conserve the environment and at the same time promote sport” [141]. BAT Kenya is also a major sponsor of the local Olympic training centre [142]. The 1994 Winter Games gold medal skier, Lina Cheryazova of Uzbekistan, was sponsored by local BAT subsidiary UZBAT AO [143].

It is perhaps in Asia that tobacco sponsorship of Olympic athletes and teams has been most active. When two Filipino athletes won medals at the 1988 Seoul Summer Games, they were rewarded prize money by the Philippines’ Tobacco Fortune Corp [144]. In 1991, the Sports Authority of Thailand and Thai National Olympic Committee pressed the government to lift restrictions on tobacco sponsorship [145]. While acknowledging that it was “illegal for us to be involved” in sponsorship, BAT subsidiary Singapore Tobacco Company (STC) described its use of “a primary sponsor as a cover” to channel funding, admitting “it needs careful handling… The politics are complex–but things are possible” [146]. A US General Accounting Office investigation of alleged violations of tobacco control policies in Asia subsequently reported that an “unnamed US cigarette company attempted to sponsor the Thai Olympic Committee” [147]. In Indonesia, BAT’s Ardath brand sponsored two gold medal badminton winners of the Summer 1992 Games, with the promotional tagline that “the players have enjoyed their own ‘taste of success” [148]. In 2000 the Chinese cigarette company Hongta Group built and financed the US$58 million Hongta Sports Centre as one of the country’s premier training complexes for the national football team in Yunnan Province [149]. In 2004, China’s largest cigarette manufacturer Baisha Group signed celebrated Olympic gold medal hurdler Liu Xiang as its “corporate image ambassador” [150]. JTI’s sponsorship of the Japanese Women’s National Volleyball Team and World Cup of Volleyball, including prominent display of its logo during global broadcasts, on team uniforms, digital scoreboards, television ads, and gift packages drew international criticism in 2011 [151].

## Discussion

This paper finds that the adoption of tobacco-free policies since 1988 have successfully restricted the most direct associations between tobacco and the Olympics. However, given the global value of the games as a marketing and promotion vehicle, the tobacco industry has continued to successfully create associations through a variety of means. It is argued that this has been due to the lack of a comprehensive policy by the IOC, which would fully detach the Olympic s from tobacco use, resulting in variation in the specific measures adopted across the 15 games held since 1988, and by individual NOCs, sports governing bodies and individual athletes.

These findings also raise important implications for realising the renewed commitment made by the IOC and WHO in 2010 for the Olympic Games to promote healthier lifestyles [6]. First, tobacco control receives only a brief mention in the IOC Marketing Fact File where “commercial associations with tobacco products… that may conflict with or be considered inappropriate to the mission of the IOC or to the spirit of Olympism” are undefined [152]. This research found LOCOG staff, for example, reportedly unaware of the tobacco-free policy and IOC Memorandum of Understanding with the WHO [6]. A genuinely tobacco-free Olympic policy should comply with Article 13 of the WHO Framework Convention on Tobacco Control (FCTC) on tobacco advertising, promotion and sponsorship. The article requires States Parties to, at a minimum, “prohibit all forms of tobacco advertising, promotion and sponsorship that promote a tobacco product by any means that are false, misleading or deceptive or likely to create an erroneous impression about its characteristics, health effects, hazards or emissions”. This includes “the use of direct or indirect incentives”, the use of “radio, television, print media and, as appropriate, other media, such as the internet,” and “tobacco sponsorship of international events, activities and/or participants therein” [153]. Further WHO guidance can be found in *A Guide to Tobacco-Free Mega Events* which calls for “absolutely no links with the tobacco industry in any form” [154]. It is recommended that the IOC and all NOCs expand the Olympic tobacco-free policy to comply with these provisions. A suggested policy is provided in Table 2.

**Table 2:** Measures for a comprehensive tobacco-free Olympics policy.

• The IOC, NOCs, sports governing bodies, teams and athletes seeking participation in the Olympic Games and related events are prohibited from receiving any form of tobacco sponsorship • All forms of tobacco advertising, promotion and sponsorship that promote a tobacco product by any means associated with the Olympics is prohibited. This includes the use of direct or indirect incentives, the use of radio, television, print media and, as appropriate, other media, such as the internet, and tobacco sponsorship of international events, activities and/or participants therein. • No tobacco products are to be distributed or sold in or near Olympic venues. • No smoking is permitted anywhere within Olympic venues including all indoor and outdoor spaces, athletes villages, and transport to and from venues. • All Olympics will include tobacco control messaging as part of broader public health campaigns to promote healthier lifestyles. • An individual with an affiliation or association with the tobacco industry is not permitted to serve in any official capacity within the Olympic movement. • Any use of the Olympic name or symbol to promote tobacco products or use is strictly prohibited.

Second, a comprehensive tobacco-free policy should be standardised and made compulsory across the IOC and all host NOCs. The current policy leaves host NOCs to decide whether to adopt tobacco-free policies at all and the specific measures to be included. As shown above, this has led to variation across different games of what precisely is meant by “tobacco free”. The voluntary nature of the policy led the Global Smokefree Partnership to write an open letter to the IOC in 2008 calling for greater leadership, and requesting the introduction of a compulsory 100% smoke-free policy for all Olympic Games [155]. Such a policy would avoid public health advocates and host NOCs being vulnerable to external pressures to adopt weaker measures such as shown in Vancouver and London.

Third, compliance with the Olympic tobacco-free policy should be extended to all NOCs, sports governing bodies, national teams and athletes seeking to participate in the Games. The Olympic Charter stipulates that NOCs “shall not associate themselves with any activity which would be in contradiction with the Olympic Charter”, and “must preserve their autonomy and resist…economic pressures.” [1] This would include composition of the local organizing committees to avoid conflicts of interest such as BAT Australia’s chairman serving on the Sydney Organizing Committee [156].

Fourth, the IOC and host NOCs should meet tobacco industry efforts to associate with the Olympics using the same vigour as the legal remedies pursued to prevent “unauthorised exploitation” of the “Olympic symbol” [157] and to protect the commercial interests of official sponsors and Olympic organisers [90,158]. For example, backed by the London Olympic Games and Paralympic Games Act 2006, LOCOG sought the removal of a flower display by a local florist and five sausage rings by a butcher to protect the “Olympic brand” [159]. Claims of a tobacco-free Olympics are directly harmed by the tobacco associations described in this paper and thus warrant similar protections.

Finally, the findings raise broader concerns about continued associations between tobacco and sports. The adoption of a comprehensive tobacco-free policy, with standardised and binding measures, would affirm the IOC’s declared commitment to promoting healthy lifestyles [6,40]. Other international and regional sporting events, such as the World Cup [160], Commonwealth Games [161] and Southeast Asian Games [162] have followed suit, and closer scrutiny of their tobacco-free policies, in light of this paper’s findings, is warranted.

## Conclusion

The adoption of Olympic tobacco-free policies since 1988 has rightfully restricted the direct use of the Games by tobacco companies to market the world’s deadliest consumer product. The findings of this paper suggest that, for the Olympics to be completely tobacco-free, a comprehensive policy based on the FCTC should be adopted and enforced throughout the Olympic movement.
